# Retraction of “Lung Cancer: MicroRNA and Target Database. Zhongguo Fei Ai Za Zhi, 2012, 15(7): 429-434”.

**DOI:** 10.3779/j.issn.1009-3419.2012.08.11

**Published:** 2012-08-20

We would retract our article from *Chinese Journal of Lung Cancer* (published in the July 2012 issue of *Chinese Journal of Lung Cancer*)^1^ due to disagreement between authors.

Challa KIRAN and Ponnala DEEPIKA

Aug, 2012

## Notes

https://www.ncbi.nlm.nih.gov/pmc/articles/PMC7969376

## References

[1] Challa KIRAN, Ponnala DEEPIKA (2012). Lung Cancer: MicroRNA and Target Database. Zhongguo Fei Ai Za Zhi.

